# Evaluating antidisease immunity to malaria and implications for vaccine design

**DOI:** 10.1111/imm.12877

**Published:** 2017-12-26

**Authors:** Temitope W. Ademolue, Gordon A. Awandare

**Affiliations:** ^1^ West African Center for Cell Biology of Infectious Pathogens Department of Biochemistry, Cell and Molecular Biology College of Basic and Applied Sciences University of Ghana Accra Ghana; ^2^ Present address: Instituto Gulbenkian de Ciência 2780‐156 Oeiras Portugal

**Keywords:** inflammation, malaria, tolerance

## Abstract

Immunity to malaria could be categorized broadly as antiparasite or antidisease immunity. While most vaccine research efforts have focused on antiparasite immunity, the evidence from endemic populations suggest that antidisease immunity is an important component of natural immunity to malaria. The processes that mediate antidisease immunity have, however, attracted little to no attention, and most interests have been directed towards the antibody responses. This review evaluates the evidence for antidisease immunity in endemic areas and discusses the possible mechanisms responsible for it. Given the key role that inflammation plays in the pathogenesis of malaria, regulation of the inflammatory response appears to be a major mechanism for antidisease immunity in naturally exposed individuals.

## Introduction

The clinical manifestation of *Plasmodium falciparum* malaria is partly mediated by direct injuries inflicted on the host by the parasite, as in the case of haemolysis of red blood cells (RBCs) during parasite egress,^1^, ^2^, ^3^ and mainly from immunopathology that ensues from inflammatory responses (acute inflammation) targeted at eliminating parasites.^4^, ^5^, ^6^, ^7^, ^8^ Therefore, protection from clinical malaria can be achieved in two key processes: antiparasite immunity, which would involve immune responses that directly suppress parasite replication and result in effective parasite clearance; and antidisease or clinical immunity, which would involve processes that altogether prevent the manifestation of clinical symptoms (i.e. immunopathology). As it takes the presence of the parasite for immunopathologies to develop, much of the research into understanding the factors that best mediate clinical immunity to malaria has been skewed to mechanisms that abrogate parasitaemia, such as antibody responses.

Humoral responses (antibody responses) have been extensively studied in the context of antiparasite immunity and clinical protection against malaria, given that the understanding of these responses can be harnessed towards the development of vaccines.^9^, ^10^, ^11^, ^12^, ^13^ These investigations are based on the premise that the induction of parasite‐specific antibodies would block important parasite processes and, as such, the cyclical replication of parasites will either be prevented or fettered.^14^, ^15^ So far, tremendous progress has been made, and several targets have been identified as vaccine candidates. In fact, a vaccine based on a liver stage antigen, the circumsporoite protein (CSP), has made it all the way to licensure (RTS,S vaccine),^16^, ^17^, ^18^, ^19^ while a handful of other antigens have been characterized and are in phases II and III clinical trials.^20^, ^21^, ^22^, ^23^, ^24^

Antiparasitic immunity, particularly antibody responses, are elicited during cumulative clinical bouts of malaria, but these responses are slowly acquired and/or are not adequately induced at levels that would confer protection in young children. As well as the problem of antigenic diversity and clonal variation on the parasites’ side, the past decade has led to the identification of several ‘faults’ that may explain why antibodies are slowly acquired, and not adequately induced in younger children. A few notable mechanisms include (i) the identification of a subset of memory B cells called ‘atypical’ memory B cells, which are inefficient at secreting antibodies^25^, ^26^ (ii) the inefficient acquisition of long‐lived plasma cells^26^, ^27^, ^28^, ^29^ (iii) the induction of an exhausted phenotype of helper T cells^30^, ^31^ and (iv) the delay in germinal centre development during infection.^32^, ^33^ Given that a vaccine, particularly blood stage vaccine candidates, would potentially rely upon an efficient humoral immune response for effectiveness in children, it is imperative to understand how malaria infection elicits these mechanisms, and possibly address whether or not an antiparasite vaccine would be successful.

Although eliminating the parasite possibly by antibody‐mediated processes is vital for protection, mechanisms that allow parasite tolerance without manifesting clinical symptoms, as observed in asymptomatic carriers (children and adults alike),^34^, ^35^, ^36^ may provide clues as to how clinical immunity (antidisease immunity) to malaria is induced. These processes have, however, attracted little attention in the malaria research community. Herein, we briefly highlight the observations that have demonstrated that, in children, humoral responses are inadequately generated *in vivo*. We emphasize the necessity for high antibody concentrations for the generation of significant *in‐vitro* growth inhibition and *in‐vivo* protection. Finally, we provide evidence of parasite tolerance at high levels of exposure: a phenomenon that hinges on the control of inflammation.

## Antidisease immunity and antiparasitic immunity to malaria: two faces of a coin

Clinical immunity to malaria infection is two‐pronged. This is because during malaria infection, two mutually inclusive processes precede pathology: (i) parasitaemia,^37^, ^38^ which leads to (ii) inflammation, including both local (as observed in cerebral malaria)^39^ and systemic inflammation.^40^, ^41^ Immunity/protection against malaria would therefore, in principle, be mediated by targeting these two key processes. The first inducer of pathology, parasitaemia, can be directly targeted by antibody‐mediated effector processes,^42^, ^43^ and this type of protection is ‘antiparasitic’ (i.e. directly targeted at reducing parasite burden). Indeed, processes that ablate humoral responses have been associated with high parasitaemia in murine malaria models, further confirming the necessity of antibody responses.^44^ Similarly, multiple longitudinal reports, such as the Garki project^45^ and others,^46^, ^47^, ^48^ have also associated high antibody levels with reduced incidence of clinical symptoms. Because the inflammatory process is also a prerequisite for pathology,^6^, ^49^ processes that culminate in reduced inflammation could potentially curb immunopathologies^50^, ^51^, ^52^, ^53^ and preclude clinical symptoms, especially against severe complications such as cerebral malaria.^53^ These processes are more targeted at directly suppressing immunopathology and, therefore, suggest an antidisease component of malaria immunity.

While both antiparasite and antidisease immunities are convoluted, and are both required to curb parasitaemia and disease manifestation, there seems to be a difference in the end‐point targets of both effector processes. Usually, high levels of antibodies correlate with reduced parasitaemias,^54^ whereas antidisease effector processes are more associated with reduced levels of proinflammatory mediators, and are strongly associated with increased prior exposures.^52^, ^55^, ^56^, ^57^, ^58^, ^59^ As immunity against clinical episodes of malaria is positively associated with increased prior exposure (or age) in endemic areas,^60^, ^61^ antidisease mechanisms (targeted at ameliorating inflammation induced pathology) might be an important mechanism mediating protection against clinical symptoms. For instance, asymptomatic carriers harbour parasites without showing clinical symptoms are mainly found at high levels of exposure^34^ and generally have lower levels of proinflammatory cytokines, including tumour necrosis factor (TNF)‐*α* and interferon (IFN)‐*γ*, than symptomatic individuals.^36^, ^62^, ^63^

This suggests that keeping inflammatory mediators low can prevent the manifestation of disease (antidisease immunity). Such a mechanism will complement the role of antibody responses (antiparasite immunity) and other effector processes that mediate parasite killing.^57^, ^64^, ^65^

## The requirements of an effective humoral response against malaria

The first insight into a role for antibodies in preventing parasitaemia and clinical malaria was seen in *Aotus* monkeys, where it was demonstrated that serum from chronically infected monkeys could potentiate malaria immunity.^66^, ^67^ Subsequently, Cohen and colleagues tested the possibility of such phenomenon in human malaria.^14^ They treated children diagnosed with malaria with purified antibodies from semi‐immune adults who resided in a hyperendemic area, and observed a marked reduction of parasitaemia and the alleviation of clinical symptoms.^14^ These independent observations showed that soluble components of serum from immune individuals confer protection against malaria, and provided the foundational evidence that led to numerous investigations into the discovery of parasite‐specific immunogens that could be developed into vaccines. However, notable facts from both experiments, which have received little attention, include: (i) the use of high concentrations of antibodies for treatment, such that in children that were cured, the passively transferred antibodies constituted up to 20% of the receiving child's total antibody titre; and when treatment failure occurred, it was associated with insufficient doses^14^, ^67^ (ii) the need for repeated passive transfer of antibodies (boosters) because protection appeared to be short‐lived, and protective effects were more pronounced when serum or antibodies were administered more than once during the course of treatment^14^, ^67^ and (iii) the most important observation from these experiments was that passively transferred serum/antibodies did not confer 100% protection, as has been observed for all characterized malaria vaccines so far, such that some participants were not cured (i.e. clinical symptoms persisted), and in participants who were cured, parasitaemia drastically reduced but was not totally cleared.^14^, ^67^ These studies revealed pertinent conditions that must be fulfilled to attain clinical immunity against malaria, namely: high antibody levels which should, preferably, be sterilizing (i.e. totally clear parasitaemia) and long‐lived. However, current evidence suggests that these requirements might be difficult to fulfil.

## Antibodies and protection: what we have learnt so far

### Insights from growth inhibitory assays

For merozoites to invade new RBCs, they must sequentially deploy several invasion ligands to engage their cognate receptors on the RBC surface.^68^ Antibodies that target these invasion ligands can therefore prevent the merozoite invasion process. Based on this premise, a panel of antigens (vaccine candidates) have been discovered and characterized. Of particular interest are those whose antibodies show strain transcending invasion inhibitory capacities. However, *in‐vitro* growth inhibition or invasion inhibition assay (GIA) results for most of these vaccine candidates demonstrate that high antibody concentrations against these antigens are required for significant growth inhibition. Furthermore, even at such high antibody levels, growth inhibition is rarely absolute. Antibodies against cysteine‐rich protective antigen (CyRPA) (full length) in two‐cycle invasion assays showed 85% inhibition at 10 mg/ml,^69^ antibodies against Rh5 (full‐length) showed 83% inhibition in a one‐cycle invasion assay at 10 mg/ml^42,70^ and a 40% invasion inhibition was observed for antibodies against Rh5 interacting protein (Ripr) (ectodomain) at 2 mg/ml in a one‐cycle assay.^42^ Similarly, antibodies against several other characterized antigens (such as MSPDBL‐1 and ‐2)^71^, ^72^ showed a comparable trend of invasion inhibition and require fairly large antibody concentrations. These observations from *in‐vitro* studies showed that high levels are required to attain considerable invasion inhibitions.

### Insights from vaccines and naturally acquired immunity

Levels of antibodies against recombinant antigens of vaccine candidates may, or may not, be detected in the plasma of children [by enzyme‐linked immunosorbent assays (ELISAs) or protein microarray] from malaria‐endemic sites in a healthy state (baseline levels), depending on age and history of exposure. For instance, levels of PfRH5 antibodies were detected in < 16% of healthy children from a Malian cohort.^43^ Similarly, the seroprevalence of antibodies to 39 *P. falciparum* antigens, including vaccine targets, varied in a Kenyan cohort, with most (96%) glycophophatidylinositol (GPI)‐anchored antigens being detected, whereas rhoptry‐associated proteins, which are mostly conserved antigens, were sparingly (5%) detected.^73^ Clinical trials of RTS,S, a malaria vaccine which targets the pre‐erythrocytic stage, have also demonstrated that vaccine recipients had higher antibody titres relative to non‐vaccinated individuals; however, protection was observed in < 40% of vaccine recipients and protection waned with time.^24^ The recently characterized *P. falciparum* sporozoite‐based malaria vaccine (PfSPZ) might provide a glimmer of hope, but similar to the pattern observed for the RTS,S vaccine, protection was also observed in 64% of vaccine recipients in a controlled human malaria infection and, particularly, antibody levels did not segregate protected from non‐protected individuals.^20^, ^74^, ^75^

Although higher antibody titres, relative to baseline levels, are generally observed during concurrent acute infection in children, several rigorous attempts to determine the specific antibody, or antibody combination, that best serve as correlate(s) of immunity have yielded disappointing results. Usually, high antibody levels are more associated with a delay to first malaria infection, and lower parasitaemias at presentation, than protection *per se*. For instance, it was observed that Rh5 responders at baseline were associated with a median 34‐day delay to the first clinical bout of malaria compared with non‐responders.^43^ This finding was confirmed in independent studies where higher Rh5 antibodies protected against high density parasitaemia, and not against re‐infection.^42^ Another longitudinal study in Ghana found a significant delay in time to first malaria infection in children with high antibody‐dependent cellular inhibition (ADCI).^76^ No evidence of protection has been found for antibodies against Ripr,^42^ while it is yet to be determined whether antibodies to CyRPA are correlates of protection. In addition, it remains to be determined whether the levels of these antibodies are within the concentrations necessary for growth inhibition as observed in GIAs, as antibody levels are often expressed as arbitrary units (AU) and/or optical densities (OD), without reflecting a metric quantification. These experiments emphasize that antibodies are indeed generated, but their levels do not seem to protect children from clinical manifestation of malaria, and emphasizes their importance in mediating protection.

Fortunately, promising synergistic inhibitory effects at comparatively lower antibody concentrations are observed when antibodies are used in combination for invasion assays, as seen in PfCyRPA–PfRh5 and PfF2–PfRH5–PfAARP–PfRH2 combinations.^70^ These *in‐vitro* findings provided evidence that buttressed the idea of a multicomponent vaccine. Several studies are now being carried out to investigate humoral responses to a large panel of antigens to determine the best combination of antigens to include in a multicomponent blood stage malaria vaccine. Osier *et al*.^73^ observed that the degree of antibody response varies, depending on the subcellular localization of the antigen in the parasite (i.e. rhoptry, microneme or surface GPI‐anchored). In addition, correlations were only observed between small proportions (24 of 648) of all pairs of antibody levels,^73^ strongly suggesting that naturally acquired immunity to a large array of antigens may not be co‐acquired. These results are also buttressed by independent experiments where the extent of correlation between antibody levels in a healthy state and during concurrent infection seemed to be antigen‐specific.^43^, ^77^ Polymorphic GPI‐anchored antigens [such as anti‐mitochondrial antibodies (AMA)‐1] often induced relatively higher antibody levels compared with conserved antigens (such as the Rhs).^43^, ^78^, ^79^, ^80^ Responses also seemed site‐specific, with different levels observed for the same antigen in different sites.^42^, ^43^ Overall, in light of this evidence, it may be difficult to determine the right combinations for a multi‐component erythrocytic stage vaccine because humoral responses to various components will differ in magnitude and kinetics, and vary based on the level of exposure.^81^ Furthermore, relatively high antibody levels do not necessarily protect children from symptomatic malaria, although they are associated with reduced parasitaemia at clinical presentation. Therefore, other mechanisms, besides humoral responses, may preclude symptomatic malaria in children exposed to perennial parasite transmission.

## The role of inflammation in malarial pathogenesis

### Malaria, a form of immunopathology

The cascade of cytokines released during the erythrocytic stages of infection, particularly pyrogenic cytokines such as TNF‐*α*, IL‐1*β* and IL‐6,^5^, ^60^, ^82^, ^83^, ^84^, ^85^ cause acute inflammation, which mediates immunopathology.^4^, ^5^, ^60^, ^86^, ^87^, ^88^ These cytokines are necessary for several antiparasitic effector processes, such as skewing the T‐cell response to a more pronounced T helper type 1 (Th1) response during infection,^89^, ^90^ directly killing or inhibiting parasite growth, as has been observed for TNF‐*α* and IFN‐*γ,* 91 and indirectly killing parasites by activating phagocytic cells.^85^, ^91^ However, if left unregulated, high levels of these cytokines, and other proinflammatory mediators have detrimental effects^57^ which manifest as fever and other symptoms of mild and severe malaria.^92^ Clearly, inflammation is at the core of the pathophysiology of malaria, and an effective control of inflammation^93^ may preclude severe disease and, perhaps, the mild clinical manifestations of malaria. Interestingly, there are data from endemic regions of Africa that show higher parasite tolerance (high parasitaemia before clinical manifestation of disease) at high exposure levels; and high parasite tolerance appears to be associated with reduced inflammatory responses.

### Evidence of increased parasite tolerance at high exposure levels

The threshold parasitaemia associated with fever (pyrogenic threshold) differs depending on the prevailing transmission intensity,^94^ such that in children from areas of low‐to‐moderate transmission intensities, symptomatic malaria is associated with comparatively lower parasitaemia^58^, ^61^, ^95^, ^96^ and fewer children harbour parasites in a healthy state (asymptomatic carriers).^34^ In the high‐transmission areas, however, this is often not the case, as higher parasite prevalence is observed in a healthy state,^95^ and most importantly higher parasitaemias are observed during clinical bouts of uncomplicated malaria.^58^, ^97^ In addition, cerebral malaria is the commonest severe form of malaria in the low transmission areas, whereas malarial anaemia is the dominant severe form in the high‐transmission areas, buttressing the notion of exposure induced clinical disparity of malaria.^96^, ^98^

This exposure‐dependent disparity of clinical and malariometric features of malaria has led to the investigation of the associated quantitative and qualitative cellular and humoral immune responses. Observations to date show that children who are highly exposed to malaria usually have higher antibody levels compared with children exposed to low‐to‐moderate transmission intensity, although antibody levels generally increase with age in both high‐ and low‐transmission areas.^26^, ^77^, ^99^, ^100^ For instance, in a Tanzanian study where altitude was a surrogate for exposure level, it was observed that levels of antibodies to merozoite surface protein (MSP)‐1_19_, MSP‐2 and AMA‐1 declined with increasing altitude (lower exposure levels).^101^ Similarly, higher seroprevalence and higher antibody titres were observed in children and adults immediately after the high‐transmission season (a period of intense exposure) relative to observed levels before the malaria season (a period of little to no transmission).^99^ Two Senegalese studies also recently reported a decline in seroprevalence of antibodies to both pre‐erythrocytic and erythrocytic stage antigens in children, as the entomological inoculation rate (EIR) declined over the years.^102^, ^103^ Clearly, high exposure levels are associated with higher antibody levels, and children harbour higher parasitaemia before manifesting clinical symptoms.^58^, ^61^, ^96^ This observation, however, raises a question: how is it that highly exposed children from endemic areas are able to prevent aggravated immunopathology which should, supposedly, ensue from comparatively higher antigenaemia (parasitaemia)? Data from recent reports suggest that increased parasite tolerance at high exposure levels is associated with reduced inflammation.

## Reduced inflammation: a potential parasite tolerance mechanism at high exposure levels

We and others^55^, ^56^, ^58^ have investigated immune responses that might promote parasite tolerance at high exposure levels. We have shown that children with malaria from low endemic areas in Ghana have higher levels of proinflammatory cytokines and higher fevers, despite relatively lower parasitaemia at presentation, compared to those who live in a high‐transmission area who present with very high parasite burdens during a clinical bout of malaria.^56^, ^58^ Although several environmental factors as well as patient autonomous factors, including age and genetics, might influence these observations, these findings are suggestive of processes that lead to reduced inflammation at high exposure levels. Similarly, semi‐immune adults who continually reside (continuous exposure) in an endemic area had lower levels of Th1 cytokines relative to levels observed in travellers experiencing their first clinical episode of malaria, and in immigrants visiting an endemic area after a long period without exposure.^104^ A controlled human model of malaria infection also observed higher natural killer cells, natural killer *γδ* T cells, and CD4^+^ IFN‐*γ* responses in naive Dutch subjects compared with observed levels in exposed Tanzanian adults from an endemic region,^105^ suggesting a control of inflammatory response with increased history of exposure.

**Figure 1:**
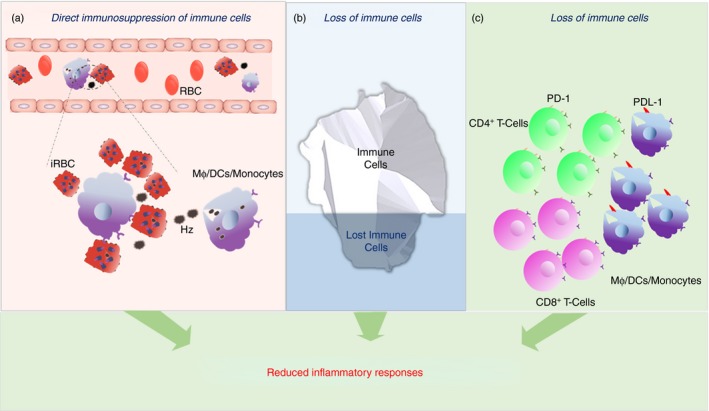
Mechanisms associated with reduced inflammatory responses at high exposure levels. (a) Direct immunosuppression of immune cells: hemozoin (Hz)‐laden immune cells [including dendritic cells (DCs), monocytes and macrophages (M*ϕ*)] exhibit impaired effector functions, such as reduced cytokine secretion and reduced expression of costimulatory molecules. (b) Loss of immune cells: repeated exposure is associated with the loss of immune cells, such as the V*δ*2 subset of *γδ* T cells, which would normally secrete high levels of interferon (IFN)‐*γ*. (c) Exhausted or refractory immune cells: T cells (including CD4^+^ and CD8^+^) may display high levels of programmed cell death 1 (PD)‐1, a marker of an exhausted phenotype, at high levels of exposure. Similarly, innate immune cells may have become refractory to stimulation by lower antigen (parasitaemia) levels, and require high antigen loads to become stimulated.

Additional studies using multiparameter flow cytometric analysis of peripheral blood mononuclear cells (PBMCs) from asymptomatic children with higher exposures revealed a skewness towards an immune‐regulatory effector phenotype of CD4^+^ T cells.^106^ A higher proportion of CD4^+^ T cells that produce IL‐10 and coproduce IFN‐*γ* and IL‐10 were observed in children from the high‐transmission area, whereas CD4^+^ T cells of children from the low‐transmission area predominantly produced IFN‐*γ*, TNF‐*α* and/or IL‐2, suggesting increased inflammatory responses in children at low exposure levels.^56^, ^106^ Similarly, CD4^+^ T cells from children with ≥ 2 prior episodes of malaria also coproduced IFN‐*γ* and IL‐10 after stimulation with infected RBCs (iRBCs), whereas CD4^+^ T cells from children with fewer than two prior malaria episodes produced TNF‐*α* only, without the co‐production of IL‐10,^107^ also demonstrating the predisposition towards an inflammatory state in children with fewer exposure histories. Furthermore, stimulation of PBMCs from children who are continually exposed to high transmission with iRBCs resulted in lower proliferation and proinflammatory cytokine secretion *in vitro*, compared to responses from children who have been historically exposed to high transmission but now live in an area of little to no exposure,^55^ also suggestive of reduced inflammatory response with continuous exposure. Taken together, these observations point to an antidisease immunity, and demonstrate that increased parasite tolerance may involve one or more mechanisms, which are yet to be clearly defined. Previous studies have, however, provided some clues which might explain the observed reduced inflammation, such as direct immunosuppression, loss and exhaustion or refractoriness of immune cells (Fig. 1a–c).

## Mechanisms of reduced inflammation at high exposure levels

### Direct immunosuppression of immune cells by infected RBCs

A possible mechanism may involve the direct immunosuppression of immune cells by parasite antigens and/or parasite products, leading to the functional impairment of immune cells. This mechanism is evident from a report where *P. falciparum* antigens induced proliferative defects in T cells.^108^ It was observed that PBMCs from infected people failed to proliferate when stimulated *in vitro* with malaria antigens, as opposed to marked proliferation that was observed in PBMCs from uninfected people, and in mitogen stimulated PBMCs from infected individuals.^109^ This proliferative defect of T cells was, however, independent of the effects of immunosuppressive T‐cell populations, and was ameliorated when parasitaemia was cleared in the patients,^109^ suggesting a direct effect of parasite antigens.

Urban *et al*.^110^ subsequently observed that DCs became functionally impaired (‘stunned’) when treated with iRBCs. This functional impairment (‘stunning’) resulted in the reduced surface expression of human leucocyte antigen D‐related (HLA)‐DR, CD54, CD40, CD80, CD83 and CD86 molecules, and consequently caused a reduced capacity of these ‘stunned’ DCs to stimulate T cells. Conversely, lipopolysaccharide (LPS)‐stimulated dendritic cells (DCs) responded appropriately.^110^ This immunomodulation of DCs by iRBCs was initially thought to depend on *P. falciparum* erythrocyte membrane protein (PfEMP)‐1;^110,111^ however, ‘stunning’ of DCs was later shown to be PfEMP‐1‐independent.^112^ Subsequent reports showed a role of hemozoin in the functional impairment of DCs and monocyte/macrophages.^108^ It was observed that hemozoin‐laden DCs expressed lower levels of the costimulatory molecule CD40, displayed impaired T‐cell stimulation and ultimately formed unstable clusters with T cells, as opposed to normal responses observed in LPS‐stimulated DCs.^113^ Moreover, *in‐vivo* studies from endemic areas showed that high numbers of hemozoin containing monocytes/macrophages were associated with decreased cytokine [IFN‐*γ* and regulated on activation, normal T cell expressed and secreted (RANTES),^114^ IL‐12^115^ and macrophage migration inhibitory factor, MIF)^116^ production, buttressing the *in‐vitro* data of hemozoin‐induced functional impairment of immune cells.

As higher parasitaemias,^58^, ^96^, ^117^ and probably higher hemozoin levels,^114^, ^116^ are observed at high exposure levels at presentation, the observed associated reduced inflammatory responses at high exposure levels may have resulted from an increased hemozoin induced functional impairment of DCs/macrophages/monocytes (Fig. 1a). In addition, it is possible that other uncharacterized parasite antigens might cause functional impairment of these immune cells.

### Loss of immune cells

The loss of T cells is perhaps the commonest observation of malaria infection in murine models and human malaria. One murine study found that 25% of memory T cells were lost in untreated mice 60 days post‐infection, compared with levels in chloroquine treated mice 34 days post‐infection.^118^ In addition, ~99% of parasite‐specific CD4^+^ T cells were lost after *P. chaubadi* infection.^119^ Another study showed that the loss of immune cells during malaria infection is unique to parasite‐specific CD4^+^ T cells, as ovalbumin‐specific T cells were not depleted in a murine malaria model.^120^ Similarly, in humans, the absolute numbers of T cells markedly increased after treatment as opposed to levels observed during concurrent infection, suggesting malaria‐induced lymphopenia.^121^, ^122^, ^123^, ^124^ Other studies found reduced numbers of T cells and natural killer cells in *P. falciparum*‐infected individuals compared with the observed numbers when these individuals were healthy,^125^, ^126^ also suggestive of infection‐induced lymphopenia.

Increased lymphopenia, particularly of inflammatory cells, may result in a reduced inflammatory response at high exposure levels. A decline in the numbers of V*δ*2 subset of *γδ* T cells was recently observed in the peripheral blood of children repeatedly exposed to malaria infection.^52^ Reduced responses, marked by lower proliferation, lower cytokine production and increased expression of immunomodulatory genes, were observed upon *in‐vitro* stimulation of these subsets of *γδ* T cells with parasite antigens. In contrast, the same subset of *γδ* T cells from children with low prior exposure histories proliferated and produced high cytokine levels, but showed lower expression of immunomodulatory genes. Gamma delta T cells have been shown to preferentially expand during malaria infection,^127^ and the V*δ*2 subset secretes high levels of IFN‐*γ* upon parasite encounter to mediate parasite killing.^52^ As IFN‐*γ* has been associated with immunopathology and severe malaria syndromes,^7^, ^128^, ^129^ decreased numbers of these IFN‐*γ*‐secreting cells would mean reduced inflammation, as has been observed at high exposure levels. The same group later reported reduced absolute numbers of forkhead box protein 3 (FoxP3)^+^ T cells in the peripheral blood of children with higher prior exposure to malaria,^130^ thus suggesting that the immunomodulation may be independent of regulatory T cells. Therefore, the predominant reduced inflammation at high exposure levels may, in fact, be as a result of loss of immune cells (Fig. 1b).

### ‘Atypical’ mechanisms of reduced inflammation

Some ‘atypical’ mechanisms responsible for reduced inflammation might be at play at high exposure levels. Perhaps immune cells have become refractory to stimulation from repeated high exposure levels, and thus require higher antigen doses (i.e. higher parasitaemia) to cause the activation of immune cells. This proposition agrees with the notion of an adaptive cellular interaction, where the activation thresholds of immune cells are being reset to higher levels following each antigen encounter.^131^, ^132^ Thus, we propose that every parasite encounter would, presumably, require a higher parasitaemia (antigen load) to stimulate and activate immune cells. This mechanism may be at play at high exposure levels, as children from these areas often present with higher parasitaemia when sick,^58^, ^61^, ^96^, ^133^ suggesting that high antigen levels are required to stimulate immune cells to produce proinflammatory cytokines, which cause immunopathology.

A parallel mechanism may involve an increased propensity of immune cells of children who reside in hyperendemic areas to become terminally differentiated, and consequently display exhausted and/or tolerized effector phenotypes.^30^ Higher levels of CD8^+^ T cells with an ‘exhausted’ phenotype have been observed during acute infections in murine models as well as human models. This exhausted phenotype is mediated by programmed cell death protein (PD‐1) (Fig. 1c), and results in CD8^+^ T cells with impaired proliferation and cytokine secretion.^31^, ^134^, ^135^ As recent findings have identified pertinent roles of CD8^+^ T cells in the clearance of erythrocytic stage parasites via different effector functions, including cytokine secretion (particularly IFN‐*γ*),^136^, ^137^, ^138^ their loss may be associated with reduced inflammatory responses at the cost of effective parasite clearance, which might explain higher parasitaemias at high exposure levels.

## Taking advantage of immune regulation

As immunoregulation seems to be at the heart of antidisease immunity, some studies have now started exploring the possibility of modulating exaggerated inflammatory responses as a therapeutic choice. For instance, the engagement of immune checkpoint molecules, such as PD‐1 by its ligand PD‐L1 (programmed death‐ligand 1), normally leads to impaired T‐cell function and expansion.^139^ This PD‐1 mediated modulation of T‐cells has been confirmed in *P. falciparum* infection,^31^, ^140^ where high expression levels were associated with reduced parasite clearance.^31^ In accordance with these observations, the combined blockade of PD‐L1 and lymphocyte‐activation gene 3 (LAG‐3) (another immuno‐inhibitory molecule) with antibodies led to increased clearance of parasitaemia, which was mediated by improved CD4^+^ T cell functions and consequently increasing antibody titers.^135^ However, another study observed T‐cell hyperactivity that promotes cerebral disease when PD‐L1 was blocked in a lethal murine model (*P. berghei*) of malaria.^141^ These conflicting studies were later corroborated by the characterization of programmed death‐1 ligand 2 (PD‐L2), another T‐cell check‐point molecule.^142^ It was elegantly shown that PD L2, but not PD‐L1, was required for both parasite clearance as well as for survival from severe diseases. Interestingly, PD‐L2 accomplished this feat by improving the Th1 CD4^+^ T‐cell response, which led to enhanced parasite clearance; and via a mechanism that increased immunomodulation, which was characterized by increased regulatory T cells.^143^ The therapeutic potential of this ligand was explored, and it was shown that soluble PD‐L2 improves survival from lethal malaria.^143^ It is yet to be determined whether repeated exposure is associated with increased levels of PD‐L2, or whether immune adults also have increased levels of PD‐L2. Altogether, this finding emphasizes that the engagement of both antiparasitic immunity and antidisease immunity are important for protection.

Another study explored the consequence of restricting the production of IFN‐*γ*, a potent inflammatory cytokine that is produced by activated terminally differentiated killer‐cell lectin‐like receptor G1 (KLRG‐1)^+^ Th1 cells during malaria infection.^144^ The authors demonstrated that signalling via the IL‐27R suppresses the development and activation of KLRG‐1^+^ Th1 cells during malaria infection by suppressing the responsiveness of these (pathological Th1) cells to IL‐12 and IL‐2 signalling.^144^ This regulation was shown to be independent of regulatory T cells, as the depletion of IL‐27R did not affect the numbers and the phenotype of FoxP3 regulatory T cells, suggesting a direct effect of the IL‐27R signalling on suppressing the activation of these pathological terminally differentiated KLRG‐1^+^ Th1 cells.^144^ These studies show that mechanisms involved in suppressing inflammatory responses might be involved in protection against malaria, and buttress our call for an increased investigation of these processes.

In a more clinically relevant context, it was observed in a randomized, double‐blind, placebo‐controlled clinical trial that treatment with Rosiglitazone led to lower levels of pyrexins, such as IL‐6 and TNF‐*α*, during concurrent malaria infection in treated patients compared with observed levels in placebo controls.^145^ The molecular mechanism(s) that explain(s) how this compound down‐modulates the release of proinflammatory mediators is yet to be deciphered; however, it is known that Rosiglitazone regulates Toll‐like receptor (TLR) signalling in immune cells,^146^ and controls (increases) the expression of CD36 in innate immune cells^146^ via cross‐regulation^147^; therefore, this might provide insights to how Rosiglitazone modulates the proinflammatory responses in immune cells. Interestingly, it was also observed in this clinical trial that the administration of Rosiglitazone led to lower parasite clearance time.^145^ This observation might be explained from another study, which showed that Rosiglitazone preferentially increases the expression of CD36 on macrophages^146^ with minimal effect on endothelial cells^148^ and this would potentially lead to increased phagocytosis by these cells.^149^ Taken together, this study shows that targeting inflammatory responses at higher exposures might indeed be protective, but these processes need to be adequately investigated. It also emphasizes the need to ensure parasite clearance, as parasite clearance and inflammatory response are intricate processes.

## Conclusion

**Figure 2:**
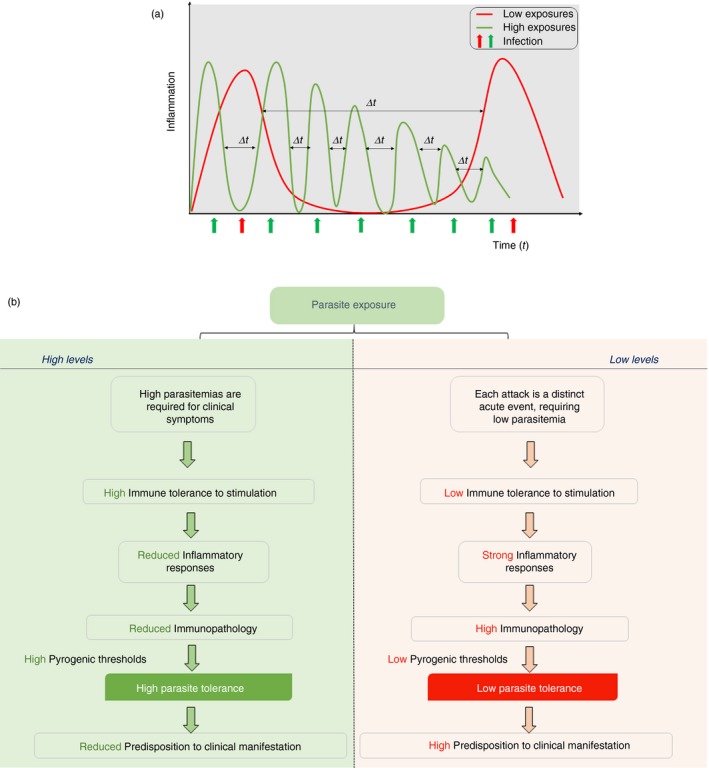
Schematic depiction of the processes leading to differences in parasite tolerance at different levels of exposure. (a) Continuous high levels of exposure mimics a chronic state of infection (low Δ*t* between infections), whereas low exposure levels are characterized by intermittent infections, separated by intervals of no exposure (high Δ*t* between infection). (b) Each infection is therefore a distinct acute event at low levels of exposure, which induce a strong inflammatory response, requiring comparatively lower parasitaemia. A strong inflammatory response is associated with increased immunopathology, and consequently results in a high predisposition to clinical symptoms. However, higher parasite densities are required to stimulate immune cells at high exposure levels (as there is usually low‐level parasitaemia without clinical symptoms), and this stimulation leads to milder inflammatory responses. Mild proinflammatory responses result in reduced immunopathology, which may preclude the manifestation of clinical symptoms.

Considering that the immunopathology from acute inflammatory responses leads to the clinical manifestation of malaria, a scenario where acute inflammation is modulated (by unknown mechanisms including those listed above) may preclude disease manifestation, even when parasitaemia persists. This might be the case in high‐transmission areas, where infections are somewhat chronic in nature, i.e. high parasite exposure in these areas favours repeated infections which mimic one chronic infection. Under this condition, the continuous exposure to parasites by immune cells leads to an increased capacity to tolerate parasite presence. Therefore, higher parasitaemia is required to activate immune cells and cause an inflammatory response that is often mild (Fig. 2b). Mild inflammatory responses consequently culminate in reduced immunopathology, which may altogether preclude the manifestation of clinical symptoms (Fig. 2a,b). Conversely, successive infections in areas with low levels of exposure would be separated by long periods of no exposure (Fig. 2a), which implies that each infection is seen by the immune system as a new acute event, and thus elicits a strong inflammatory response (Fig. 2a). As such, parasite tolerance is lower compared to levels observed at high‐transmission areas. Altogether, strong inflammatory responses cause immunopathology, which results in a high predisposition to clinical symptoms (Fig. 2b).

These observations have important implications for vaccine efficacy studies and future vaccine designs, particularly vaccines that prevent exposure to blood stage parasites altogether. For instance, RTS,S, the most advanced malaria vaccine, was associated with serological evidence of reduced exposure (reduced breadth and magnitude of antibodies),^150^ suggesting a scenario where exposure to blood‐stage parasites was ‘knocked‐down’ among the vaccinated cohort. Considering that vaccinated children were shown to be at higher risk of clinical malaria at the end of the study compared to unvaccinated ‘wild‐type’ children,^24^ it is possible that vaccine‐induced loss of exposure to blood stage parasites resulted in a loss of immune tolerance. Consequently, vaccinated children were at a higher risk of clinical malaria at the end of the study, presumably because they were unable to control inflammation as much as the unvaccinated children.

Further work is required to clearly elucidate the mechanisms of parasite tolerance leading to clinical immunity. Ideally, such studies should be longitudinal; for example, using a birth cohort to investigate the evolution of inflammatory responses as well as adaptive immunity from the first infection of the malaria‐naive infant until acquisition of clinical immunity.

## Author contributions

The paper was written by TWA and GAA. GAA critically reviewed the paper and provided mentorship to TWA.

## Funding statement

TWA and GAA were supported by funds from a DELTAS Africa grant (DEL‐15‐007: Awandare) and the Royal Society of Tropical Medicine and Hygiene Small Grant (GR000775: Ademolue). TWA was also supported by a Master's fellowship from a World Bank African Centres of Excellence grant (ACE02‐WACCBIP: TWA). The DELTAS Africa Initiative is an independent funding scheme of the African Academy of Sciences (AAS)'s Alliance for Accelerating Excellence in Science in Africa (AESA) and supported by the New Partnership for Africa's Development Planning and Coordinating Agency (NEPAD Agency) with funding from the Wellcome Trust (107755/Z/15/Z: TWA) and the UK government. The views expressed in this publication are those of the author(s) and not necessarily those of AAS, NEPAD Agency, Wellcome Trust or the UK government.

## Disclosures

The authors declare no competing interests.
